# Correlation of Plasma MMP-1 and TIMP-1 Levels and the Colonic Mucosa Expressions in Patients with Ulcerative Colitis

**DOI:** 10.1155/2009/275072

**Published:** 2009-11-09

**Authors:** Ying-De Wang, Xiao-Yan Tan, Ke Zhang

**Affiliations:** ^1^Department of Gastroenterology, The First Affiliated Hospital of Dalian Medical University, Dalian, Liaoning 116011, China; ^2^Department of Gastroenterology, Affiliated Xinhua Hospital of Dalian University, Dalian, Liaoning 116023, China

## Abstract

*Background*. Both plasma and mucosal levels of matrix metalloproteinase-1 (MMP-1) and tissue inhibitor of metalloproteinase-1 (TIMP-1) have been shown to be independently correlated with ulcerative colitis (UC), but their relationship with each other and to disease severity remains unclear. This study aims to evaluate the relationship between colonic mucosal and plasma levels of MMP-1 and TIMP-1 with each other and with the severity of ulcerative colitis (UC). *Methods*. Colonic mucosal lesions and venous blood samples were collected from 30 patients with UC and 15 normal subjects. Real-time reverse transcription-PCR and immunohistochemistry were used to determine colonic mucosal MMP-1 and TIMP-1 expression; ELISA was used to measure plasma levels of MMP-1 and TIMP-1. *Results*. Expression of colonic mucosal and plasma MMP-1 and TIMP-1 in patients with UC was significantly higher than that of controls (*P* < .05), and was positively correlated with disease severity (*P* < .05). Plasma MMP-1 and TIMP-1 levels were well correlated with their corresponding expression in colonic mucosa (*P* < .05, *r* = 0.805 and 0.908). *Conclusion*. Plasma MMP-1 and TIMP-1 levels reflect their colonic mucosal expression to some extent in patients with UC. Plasma MMP-1 and TIMP-1, in particular, demonstrate the potential to become biomarkers to clinically diagnose UC, predict its severity, and guide further therapy.

## 1. Introduction

Ulcerative colitis (UC) is a chronic nonspecific inflammatory disease of the colon with unknown etiology. No specific effective treatment is available at present. Pathological colonic lesions are usually limited to the mucosal and submucosal areas, while diarrhea, pusmucus bloody stool, and abdominal pain are common clinical symptoms.

More attention is being focused on this disease because its morbidity has increased in recent years in China, where UC was once an uncommon gastrointestinal disorder. Previous studies on the pathogenesis of UC have shown that colonic mucosal inflammation and ulceration are closely related to the excessive degradation of extracellular matrix (ECM) by matrix metalloproteinases (MMPs), which are overexpressed and activated to cause colonic mucosal injury and inflammation [1]. MMPs are a group of zinc-dependent proteases that are produced and secreted by connective tissue cells, endothelial cells, mono-macrophages, and other cells. Their activity can be inhibited by their natural inhibitors—tissue inhibitor of metalloproteinases (TIMPs). TIMPs are a group of secretive glycoproteins that have the capacity to inhibit MMP activity, thereby attenuating ECM degradation. TIMPs also participate in tissue structural modeling and maintenance, and indirectly influence ECM-dependent signal transduction. Four subtypes of TIMPs have been identified in mammals: TIMP-1, TIMP-2, TIMP-3, and TIMP-4. TIMP-1 is a soluble glycoprotein with a molecular weight of 29 kD that inhibits primarily MMP-1, MMP-3, and MMP-9.

Animal and clinical studies have revealed the overexpression of various MMPs and TIMPs in the inflammatory areas of colonic mucosa in UC; MMPs expression tend to be particularly high. It is believed that increased ratio of MMP/TIMP is one of the mechanisms in the pathogenesis of UC [2–4]. MMP-1 and TIMP-1 are thought to be more closely associated with UC than other MMPs and TIMPs; studies [2, 5–7] have reported that their expression in patients with UC is significantly higher than in normal control subjects. In particular, MMP-1 overexpression has been correlated with mucosal inflammation and the initial steps of ulceration. Furthermore, MMP-1 and TIMP-1 levels in the peripheral blood were also found to be elevated in patients with UC in studies by Wiercinska-Drapalo [8] and Holten-Andersen [9]. However, the correlation between plasma levels of these proteins and their mucosal expression or with UC disease severity is not clear. In this study, we examined MMP-1 and TIMP-1 expression both in the colonic mucosa and plasma to study their relationship and association with disease severity in patients with UC.

## 2. Materials and Methods

### 2.1. Patients and Samples

Thirty patients (male, *n* = 14; female, *n* = 16; mean age, 46 years; age range, 23–73 years) with UC diagnosed by clinical symptoms, endoscopy, and pathology findings were enrolled in the study. Of the 30 patients, 4 patients had pan-colon lesions, 2 had semicolon lesions, 17 had rectosigmoid lesions, and 7 had rectal lesions. Patients were divided into groups based on the diagnostic criteria of UC severity: 12 patients were classified as mild (*M* group) and 18 classified as moderate-to-severe (MTS group). Meanwhile, 15 normal subjects were recruited as normal controls (male, *n* = 6; female, *n* = 9; mean age, 41 years; age range, 22–63 years).

Biopsy samples from mucosal lesions of UC patients and normal mucosa of the normal control subjects were divided into two parts. One part was immediately snap frozen in liquid nitrogen and stored at −80°C for reverse transcription- (RT-) PCR, and another part was fixed in formalin, embedded in paraffin, and cut into 4 *μ*m thick sections for immunohistochemistry.

Blood was also drawn from both groups. The supernatant was collected after spinning at 1000 × g for 30 minutes, and then at 10 000 × g for 10 minutes at 2°C to 8°C to remove blood platelets. Samples were stored at −80°C for ELISA.

### 2.2. Total RNA Extraction

Total RNA was extracted from the frozen samples using an RNA isolation kit (Invitrogen, Calif, USA) following the manufacturer's instructions. RNA integrity was assessed on a 1% agarose gel.

### 2.3. RT-PCR for MMP-1 and TIMP-1

RT-PCR was performed using the TaKaRa RNA PCR kit 3.0 (AMV; Dalian Baosheng Biotechnology Company) following the manufacturer's instructions. Primer sequences used in this study were as follows:

MMP-1:

sense: 5′-ATGCGAACAAATCCCTTCTACC-3′;

antisense: 5′-TTCCTCAGAAAGAGCAGCATCG-3′;

TIMP-1:

sense: 5′-GGACACCAGAAGTCAACCAGACC-3′;

antisense: 5′-CGTCCACAAGCAATGAGTGCC-3′.

*β*-actin (internal control):

sense: 5′-CCTTCCTGGGCATGGAGTCCTG-3′,

antisense: 5′-GGAGCAATGATCTTGATCTTC-3′.

Reverse transcription was carried out at 30°C for 10 minutes, 42°C for 30 minutes, 99°C for 5 minutes, and 5°C for 5 minutes. PCR conditions were as follows: initial denaturation at 94°C for 2 minutes, followed by 35 amplification cycles of 94°C for 30 seconds, 53°C for 30 seconds, at 72°C for 1 minute, and extension at 72°C for 10 minutes.

For semiquantitative analysis of gene expression, amplification products were assessed on a 2% agarose gel. The band density was determined by the Bio-imaging system (PALL Company, USA). MMP-1 mRNA and TIMP-1 mRNA were expressed by the ratios of MMP-1, TIMP-1, and *β*-actin OD values.

### 2.4. Immunohistochemistry

After initial treatment, samples were incubated with the primary antibodies, mouse antihuman MMP-1 monoclonal antibody and mouse anti-human TIMP-1 monoclonal antibody (Santa Cruz, USA), at room temperature for approximately 1.5 hours. Samples were washed 3 times with PBS, then incubated with a peroxidase-conjugated secondary antibody for 15 minutes, followed by washing with PBS. Samples were incubated with the substrate diaminobenzidine (DAB) for 10 minutes.

Positive results of immunohistochemistry were determined by the appearance of brown or yellow DAB staining. The Image-Pro-Plus 4.5 microscopic image analyzing system was used to determine the intensity of staining. Five fields in each section were randomly chosen, and the total optical density and total image area were measured. The mean optical density was calculated as the ratio of the total optical density and total image area; larger mean optical densities indicate greater expression of the corresponding proteins.

### 2.5. ELISA

ELISA for plasma MMP-1 and TIMP-1 was performed using an ELISA kit (R&D Systems, USA) following the manufacturer's instructions. Protein concentrations were determined using a standard curve.

### 2.6. Statistical Analysis

All values were expressed as mean ± SD. Analyses of variance (ANOVA) with Student-Neuman-Keuls post-hoc test were used to compare mRNA levels of MMP-1 and TIMP-1 in patients with different disease severity. Spearman correlation analysis was used to determine the relationship between plasma and mucosal MMP-1 and TIMP-1 levels. *P* < .05 was considered statistically significant. All statistical analysis was performed using SPSS 11.5 for Windows.

## 3. Results

### 3.1. MMP-1 and TIMP-1 Expression in Colonic Mucosa by RT-PCR

Colonic mucosal expressions of MMP-1 and TIMP-1 in UC patients were significantly higher than those of in the normal controls (*P* < .05, Table 1). In addition, MMP-1 and TIMP-1 expression increased with the severity of ulcerative colitis (Table 1).

### 3.2. MMP-1 and TIMP-1 Expression in Colonic Mucosa by Immunohistochemistry

**Figure 2:**
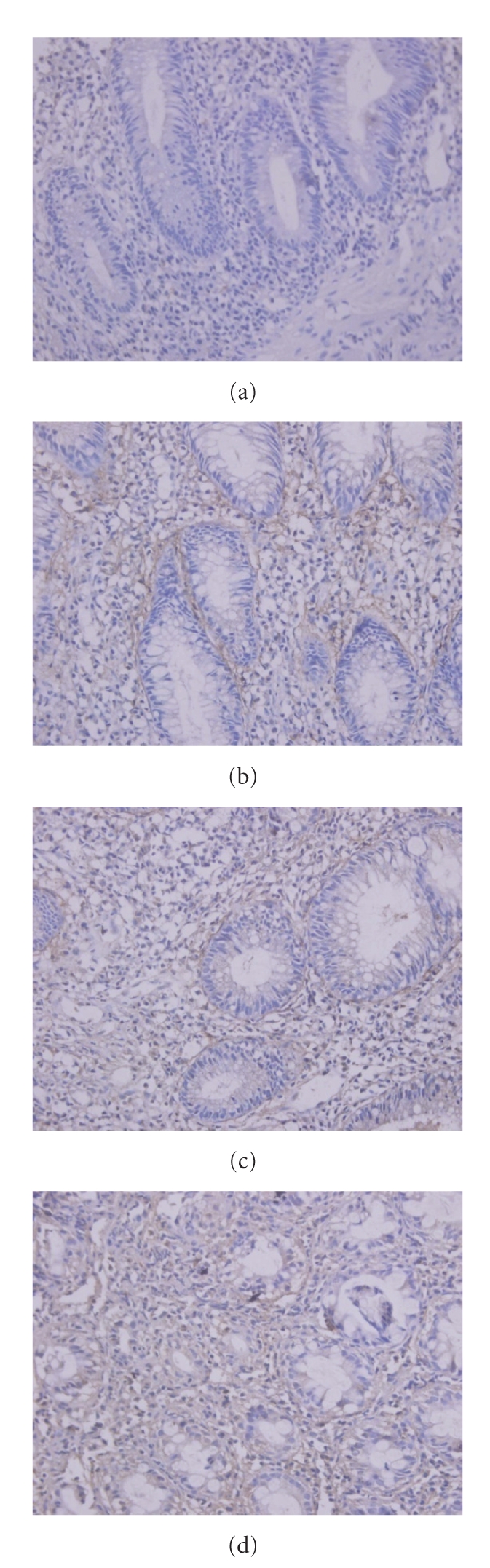
Immunohistochemistry showing MMP-1 expression in (a) control group, (b) *M* group, and (c, d) MTS group: *M*, mild; MTS, moderate-to-severe.

Similar results were obtained with immunohistochemistry. MMP-1 and TIMP-1 expression levels in both the mild (*M*) and moderate-to-severe (MTS) groups were significantly higher than those of normal controls (*P* < .05), and they were expressed to a greater extent in MTS group compared to *M* group (*P* < .05; Table 1; Figure 2).

### 3.3. MMP-1 and TIMP-1 Blood Level by ELISA Method

The ELISA study (Table 1) showed that the overall plasma levels of MMP-1 and TIMP-1 in UC patients were significantly higher than those of the control group (*P* < .05). However, no difference was observed between plasma MMP-1 levels of patients with mild ulcerative colitis and normal controls or between disease severity groups (MTS versus *M* group; *P* > .05). In contrast, plasma TIMP-1 levels in the MTS group were significantly higher than those of the *M* group and significantly higher in the *M* group compared to the normal controls (*P* < .05).

### 3.4. Correlation Analysis of Plasma and Mucosal MMP-1 and TIMP-1 Levels

**Figure 3:**
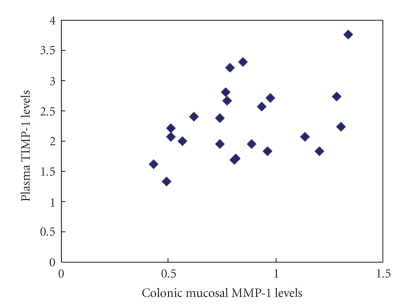
Correlation between plasma and mucosal MMP-1 expression.

**Figure 4:**
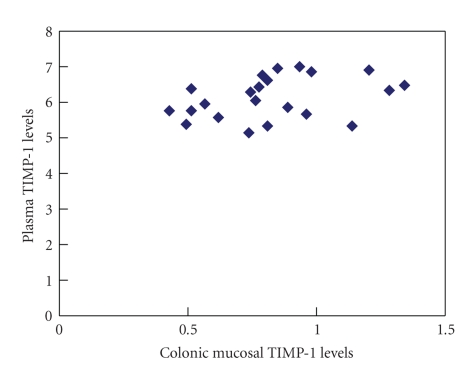
Correlation between plasma and mucosal TIMP-1 expression.

Plasma MMP-1 and TIMP-1 levels were correlated with their colonic mucosal expression in UC patients (MMP-1, *P* < .05, *r* = 0.81; TIMP-1, *r* = 0.91; Figures 3 and 4).

## 4. Discussion

In this study, we determined MMP-1 and TIMP-1 expression levels in ulcerative colitis patients compared with normal controls. Colonic mucosa expression was determined at the mRNA and protein levels by RT-PCR and immunohistochemistry. The corresponding plasma levels of MMP-1 and TIMP-1 were determined using ELISA. Both MMP-1 and TIMP-1 expression increased in the colonic mucosa of UC patients compared to normal controls, and the expression levels were correlated with disease severity. Similar findings were found in the analysis of plasma. Plasma levels of both MMP-1 and TIMP-1 correlated well with their colonic mucosa levels. However, plasma TIMP-1 was well correlated with disease severity, but plasma MMP-1 was not.

 Our results are consistent with findings from McKaig et al. [10] and supported previous studies demonstrating that MMP-1 reflected acute tissue injury and was associated with the initial steps of ulceration in UC and new blood vessel formation. [2, 4] Von Lampe B et al. [1] also showed that TIMP-1 mRNA expression was 9- to 12-fold increased in UC lesions.

MMP and TIMP expression increased not only in tissues but also in the peripheral blood samples from patients with acute coronary syndrome, cancer, hepatitis, rheumatoid arthritis, and normal persons.[11–15] Wiercinska-Drapalo A et al. [8] used ELISA to determine MMP-1 and TIMP-1 expression and found that serum MMP-1 and TIMP-1 were significantly increased in patients with UC compared with normal controls. They also found that TIMP-1 level was related to endoscopic mucosal injury, disease activity index, clinical activity index, and C protein level although MMP-1 was not. Our study produced similar findings; plasma MMP-1 levels were higher in UC patients, but did not correlate with disease severity. The plasma and mucosal TIMP-1 levels were better indicators of disease severity. Further analyses of plasma levels and mucosa expression revealed that plasma levels of both MMP-1 and TIMP-1 correlated with mucosa expression. These findings indicate that plasma MMP-1 and TIMP-1 generally reflect the disease state, and TIMP-1 level was additionally associated with disease severity.

MMP-1 is primarily associated with the initial steps of ulceration in UC, [2, 4] which may explain its lack of association with disease severity. In our study, most of the patients were suffering from chronic recurrent UC, so plasma MMP-1 could have been affected. The ELISA assay used in this study measures only free MMP-1 and MMP-1 combined with TIMP-1. Thus MMP-1 combined with *α*_2_-macroglobulin could not be detected. In addition, TIMP-1 is more easily released into the blood; [15] therefore, its measurement sensitivity is higher than that of MMP-1. According to a previous study, [16] free MMP-1 decreased and MMP-1 combined with TIMP-1 increased as the disease progressed. This may be another reason why plasma MMP-1 level increased only in moderate-to-severe disease patients, but not in mild disease patients.

In conclusion, plasma MMP-1 and TIMP-1 reflect their colonic mucosal expression to some extent; plasma TIMP-1 reflects colonic mucosa lesion severity more sensitively than plasma MMP-1 level. After excluding other diseases such as coronary heart disease and liver disease that are also associated with increased MMP-1 and TIMP-1 expressions, plasma MMP-1 and TIMP-1, especially TIMP-1, could possibly become useful biomarkers for UC diagnosis and disease severity, and may be useful to guide therapy.

## Figures and Tables

**Figure 1:**
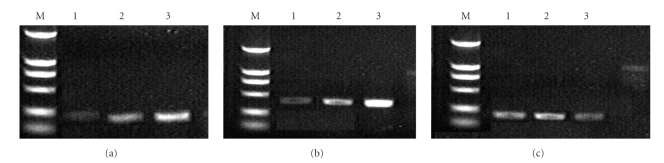
RT-PCR results for MMP-1(A), TIMP-1(B), and *β*-actin(C): *M*, marker, lane 1, normal controls; lane 2, *M* group; lane 3, MTS group.

**Table 1:** MMP-1 levels in colonic mucosa and plasma.

	Controls	UC	Mild	Moderate-to-severe
	(*n* = 12)	(*n* = 30)	(*n* = 12)	(*n* = 18)
**Colonic mucosa**				
RT-PCR				
* *MMP-1	0.27 ± 0.04	0.63 ± 0.23*	0.46 ± 0.08*	0.80 ± 0.19*^^^
* *TIMP-1	0.51 ± 0.08	0.92 ± 0.24*	0.75 ± 0.14*	1.06 ± 0.22*^^^
Immunohistochemistry				
* *MMP-1	0.005 ± 0.001		0.075 ± 0.003*	0.081 ± 0.003*^^^
* *TIMP-1	0.013 ± 0.003		0.080 ± 0.003*	0.090 ± 0.006*^^^
**Plasma **				
ELISA				
* *MMP-1	1.90 ± 0.37	2.46 ± 0.60*	2.21 ± 0.40	2.68 ± 0.68
* *TIMP-1	5.59 ± 0.30	6.37 ± 0.54*	6.14 ± 0.48*	6.60 ± 0.50*^^^

*P < .05 compared to controls ^^^ *P* < .05 *M* group
